# Uncertain Times: A Continuing Care Retirement Community's COVID-19 Communication Response

**DOI:** 10.1093/geroni/igab046.2235

**Published:** 2021-12-17

**Authors:** Justine Sefcik, Martha Coates, Minjung Shim, Don McEachron, Rose Ann DiMaria-Ghalili, Kathleen Fisher

**Affiliations:** 1 Drexel University, College of Nursing and Health Professions, Philadelphia, Pennsylvania, United States; 2 Drexel University, Bryn Mawr, Pennsylvania, United States; 3 Drexel University, Philadelphia, Pennsylvania, United States

## Abstract

The purpose of this qualitative inquiry was to explore conversational video recordings by top administrators from a faith based Continuing Care Retirement Community (CCRC) to help residents, staff, and family members manage the associated uncertainties of the pandemic. Six interdisciplinary researchers explored 37 video communiques from March 2020 to February 2021. Data was independently coded using latent content analysis with the team building consensus on major themes. Themes identified were: Building Trust through Transparency, We’re in this Together, Power of One/Individual Responsibility, Converting Challenges into Teaching Moments, and Gratitude/Resilience. Findings suggest attempts to inform, reassure, and encourage maintaining a safe environment (e.g., using masks, restricting visitors, vaccine promotion) for residents, staff, and family members, was met through these conversational videos. Leadership of this CCRC exemplified the mission to provide transparent information during the pandemic, serving as a model to inform other CCRCs’ communication during this pandemic and other crisis situations.

